# Is provisioning rate of parents and helpers influenced by the simulated presence of novel individuals?

**DOI:** 10.1007/s00265-024-03548-2

**Published:** 2025-01-15

**Authors:** Pietro B. D’Amelio, Nora V. Carlson, Arnaud Tognetti, Marina Sentís, Liliana R. Silva, Fanny Rybak, Rita Covas, Claire Doutrelant

**Affiliations:** 1 CEFE, CNRS, Univ Montpellier, EPHE, IRD, Montpellier, France; 2 FitzPatrick Institute of African Ornithology, University of Cape Town, 7701 Rondebosch, South Africa; 3 Université Paris-Saclay, CNRS, Institut des Neurosciences Paris-Saclay, 91400 Saclay, France; 4 Consiglio Nazionale delle Ricerche—Istituto di Ricerca sulle Acque (CNR-IRSA), Brugherio, Italy; 5 Juanes Lab, Department of Biology, University of Victoria, Victoria, BC Canada; 6 CEE-M, University of Montpellier, CNRS, INRAE, Institut Agro, Montpellier, France; 7 Terrestrial Ecology Unit, Department of Biology, Ghent University, Ghent, Belgium; 8 Centro de Investigação em Biodiversidade e Recursos Genéticos, Laboratório Associado, CIBIO-InBio, University of Porto, Campus Agrário de Vairão, 4485-661 Vairão, Portugal; 9 BIOPOLIS Program in Genomics, Biodiversity and Land Planning, CIBIO, Campus de Vairão, 4485- 661 Vairão, Portugal

**Keywords:** Audience effects, Cooperation, Direct benefit, Food provisioning, Partner choice, Playback

## Abstract

**Abstract:**

Cooperative behaviour is widespread in animals and is likely to be the result of multiple selective pressures. A contentious hypothesis is that helping enhances the probability of obtaining a sexual partner (i.e., confers direct benefits through sexual selection). Under this hypothesis, cooperative behaviours may have evolved into a signal. Consequently, we would expect individuals to enhance cooperation when a potential mate is present, to signal their status and quality. We evaluated this possibility in the cooperatively breeding sociable weaver (*Philetairus socius*). We simulated the presence of different types of individuals using a playback to test whether the simulated presence of an unknown individual, possibly a potential mate, increases provisioning rate in two classes of cooperating birds : breeders and helpers. If the signal is the provisioning rate in itself we expected increased feeding rates of male helpers during the simulated presence of an unknown female. Contrary to our predictions, the simulated presence in the audience of an unknown individual did not influence the nestling provisioning rate of birds of any sex and class. From these results, we conclude that in this species the variation in provisioning rate is unlikely to be used as a signal in a sexual selection context. However, we also highlight the limitations of our methods and suggest improvements that future studies should incorporate when testing audience effects on cooperation.

**Significance statement:**

Animals may cooperate to gain direct benefits, like attracting mates. This happens for example in humans. In species where cooperation leads to direct sexual benefits, when the appropriate audience is present, (i.e., a potential mate), helpers should enhance their cooperation. To determine whether helping to raise others’ young varies according to who is watching, we used playbacks to simulate the presence of unknown individuals of opposite sex (potential mates) while helpers were feeding young. Helping, quantified here as number of times food was brought to the chicks over an hour, was not affected by the simulated audience. We concluded that in sociable weavers variation in provisioning rate is unlikely to be a signal to obtain direct sexual benefits.

**Supplementary Information:**

The online version contains supplementary material available at 10.1007/s00265-024-03548-2.

## Introduction

Care for young by individuals other than parents is a central feature of many cooperative systems. Understanding the selective pressures underlying the evolution and maintenance of cooperative care has been a main focus within ecology and evolution (reviewed in Cockburn 1998; West et al. 2007, 2021). Though cooperation is generally well explained by kin selection providing indirect benefits (Hamilton 1964; Kay et al. 2020), this does not preclude several direct benefits from (co-)occurring (Clutton-Brock 2002; Covas and Doutrelant 2019).

In the context of food provisioning of offspring, possible direct benefits of helping have been hypothesised to occur due to multiple mechanisms. Under the “group augmentation” hypothesis, cooperatively breeding animals are expected to increase the overall reproductive success of the group and increased group size is expected, in return, to improve survival or future reproductive success (Kokko et al. 2001; Kingma et al. 2014). Other hypotheses rely on the benefits obtained through the behaviour of other individuals in response to the helper’s cooperative actions. In particular, the “pay to stay” hypothesis proposes that cooperating individuals help to assure their access to the group’s communal resources (Gaston 1978; Kokko et al. 2002; Bergmüller and Taborsky 2005; Zöttl et al. 2013). The “partner choice” hypothesis (Taborsky et al. 2016; Covas and Doutrelant 2019), also referred to as “social prestige” (Zahavi 1995; Wright 1999; Bergmüller et al. 2010), suggests that helping leads to a preference for more cooperative individuals during sexual or social partner choice. These hypotheses suggest that helping reflects individual quality or propensity to cooperate in the future and that bears an individual cost (e.g., condition, Russell et al. 2003; Covas et al. 2022) which could result in survival costs, and therefore may act as cues or signals that provide information about an individual’s characteristics, quality and/or condition (Gintis et al. 2001; Covas and Doutrelant 2019). This mechanism is analogous to sexual mate choice that usually relies on the assessment of specific display behaviours. Courtship displays, for example, take many forms such as singing or dancing, which often bear a cost for the performer, and are generally thought to be honest signals of fitness quality (Johnstone 1995; Dougherty 2021). Under the pay to stay and partner choice hypotheses, it may be beneficial for helpers to display their cooperative behaviour by exaggerating some features of the provisioning behaviour, in the presence of the target audience.

Signallers are sensitive to the target audience’s presence (McGregor 2005). For instance, roosters call more frequently when they find food in the presence of hens (Marler et al. 1986), male zebra finches (*Taeniopygia guttata*) sing faster when with females (Sossinka and Böhner 1980) and, in a cleaner-client fish mutualism, cleaners (*Labroides dimidiatus*) are more cooperative when there is a client audience (Pinto et al. 2011). This behavioural flexibility is referred to as “audience effects” (Marler et al. 1986) and describes how expression of many animal behaviours depends on the presence and identity of observers, an effect which is found across several contexts and taxa (Zuberbühler 2008). Many animal signals are therefore expected to be produced strategically, with signallers adjusting their output in response to a specific audience. If individuals use cooperative behaviour as a signal, we then expect that cooperators (i.e. individuals engaging in cooperative behaviours) will express those behaviours differently when their target audience (e.g., the potential mates or breeders) is present to maximize signal efficiency and therefore potential gains (McDonald et al. 2008b). Moreover, to quantify if, when and how cooperators change their behaviour according to the audience is fundamental to understanding the evolution and maintenance of cooperative systems (Nowak and Sigmund 2005). Cooperative behaviours could be used as signals directed towards social or sexual partners (Covas and Doutrelant 2019). Indeed, cooperative behaviours being used as signals in a social context has been shown in several species, including humans (Milinski et al. 2002; Bshary and Grutter 2006; Zöttl et al. 2013; Schlaepfer 2018). However, in the context of mate choice, evidence that audience effects increases cooperative behaviour, has only been found in humans, with some studies showing that men are more cooperative in the presence of women (Tognetti et al. 2012; Van Vugt and Iredale 2013; Raihani and Smith 2015; for critical review, see Bhogal et al. 2019). Whether similar audience effects are found in other cooperative animals remains insufficiently studied (Covas and Doutrelant 2019), and the influence of mate choice on cooperative behaviour in animals is poorly understood.

Audience effects have previously been used in cooperatively breeding species to investigate whether helping-at-the nest can be used as a signal. For example, studies in cooperatively breeding cichlid fish (*Neolamprologus pulcher*) have found good support for a pay-to-stay mechanism (Bruintjes and Taborsky 2008). In birds, most studies investigated the showiness of helping behaviour by assessing whether feeding events were synchronised with the arrival of the presumed receivers of the signal within their breeding groups (i.e., what was considered to be the relevant audience), which were either the breeders (under the pay-to-stay hypothesis) or potential partners (under a partner choice mechanism). In contrast to the results on cichlids, experimental studies in birds have repeatedly failed to find support for a signalling mechanism whether related to pay-to-stay or partner choice (McDonald et al. 2008b; Nomano et al. 2013; Koenig and Walters 2016; reviewed in Covas and Doutrelant 2019). Exceptions were two observational studies on carrion crows *Corvus corone* and sociable weavers, and anecdotal reports have also been observed in Arabian babblers *Turdoides squamiceps* (Carlisle and Zahavi 1986). In carrion crows subordinate females matched their arrival at the nest during provisioning with dominant breeders in accordance with a pay to stay mechanism (Trapote et al. 2021). In sociable weavers helpers carrying food to the nestlings waited longer than parents at the colony before entering the nest to feed, but went into the nest more rapidly if in the presence of a larger audience (Doutrelant and Covas 2007). In addition, helpers spent more time at the colony with prey before feeding the nestlings when they brought more food and when rainfall was lower (i.e. when food is potentially harder to find; Doutrelant and Covas 2007). This was interpreted as an indication that helpers behaved in a way that maximised their chances of being seen feeding at the nest, and therefore that helping-at-the-nest in this species had signalling characteristics.

Sociable weavers live in large colonies (up to several hundred individuals; Maclean 1973a) that integrate both kin and non-kin individuals (Covas et al. 2006; van Dijk et al. 2014), and behaviours can be easily observed by both breeding group members and other non-kin individuals. Additionally, the colonies can be visited by prospecting birds (both females and males, RC and CD, unpublished data) and immigration and formation of new breeding pairs can occur all-year round (D’Amelio et al. 2024). Consequently, an individual engaging in cooperative behaviour may be able to gain benefits from signalling outside the breeding group (e.g., to prospecting individuals). In contrast, in most studies conducted previously, the targets of possible signals were considered to be the individuals within the breeding group (bell miners *Manorina melanophrys* (McDonald et al. 2008a), acorn woodpecker *Melanerpes formicivorus* (Koenig and Walters 2016), and chestnut-crowned babbler *Pomatostomus ruficeps* (Nomano et al. 2013). We therefore consider that in sociable weavers nestling feeding could be used as a signal by male helpers to attract potential mates through signalling their nestling feeding ability to prospecting females. Both males and females can help their parents during their first year. However, males often stay as helpers until they find a partner, usually an immigrant, while females disperse to breed in other colonies (van Dijk et al. 2014), rarely becoming helpers after dispersing. Hence, helpers older than one year are more often males than females (Doutrelant et al. 2004; 72%, A.C. Ferreira et al. unpubl. data). Individuals can start helping from a very young age (as early as 56 days in the present study), several months earlier than the youngest recorded breeder (237 days, D’Amelio et al. 2024). Helpers very rarely sire offspring in the brood they are helping (RC and CD unpublished data, 1.1% in the present study, *N* = 92) and although helpers are most often non-breeders (i.e., do not have a partner yet), some individuals who have already had a failed breeding attempt during the season will become a helper within the same season. However, these instances of dual roles are very rare (< 5% of breeders, RC and CD unpublished data).

Here, we address the question of whether nestling provisioning rate is influenced by audience effects in sociable weavers. To test the hypothesis that helpers increase nestling feeding rate in the presence of an unknown individual of the opposite sex (considered to be a potential mate, hence in the context of mate-choice), we simulated the presence of unknown male and female birds using playbacks. Sociable weavers are long-term monogamous and breeders very rarely divorce or have extra-pair offspring (D’Amelio et al. 2024), thus we expect breeders not to respond to the playback of novel individuals. In contrast, if male helpers use helping-at-the-nest as a signal to prospecting females, then we predict the following:

1. Feeding rates of helper males would increase in the presence of unknown female calls, while the feeding rates of female helpers and breeders would not change.
2. Only unknown female’ calls would receive a response, while control and unknown male calls would be ignored by both sexes of breeders and helpers.
3. That older helper males would be more responsive (i.e. more likely to increase their feeding rate) than younger helper males as older males are more likely to be of breeding age.

## Materials and methods

### Study species and field site

#### Sociable weavers

Sociable weavers are colonial facultative cooperative breeders (Covas et al. 2008). They build and live in large communal nest colonies within which groups and breeding pairs use individual nest-chambers for roosting and breeding throughout the year (Maclean 1973a). The nestling period lasts up to 25 days (Maclean 1973b). The breeding pair (i.e. breeders) can be assisted in raising the offspring by other individuals (i.e. helpers) in groups that range from 2 to 10 individuals (Maclean 1973b; D’Amelio et al.^2024^). Helpers participate in all parental activities (provisioning, nest chamber maintenance and defence), but usually feed less than the breeding pair (Doutrelant and Covas 2007; A.C. Ferreira et al. unpubl. data). Sociable weavers’ helpers are usually close relatives of the breeding couples but 14.3% were found to be unrelated (*R* ≤ 0.125, A.C. Ferreira et al. unpubl. data).

#### Study site

The study site is located within Benfontein Nature Reserve in the Northern Cape Province of South Africa (28°520 S, 24°500E, ~ 15km^2^, 1,180 m). Most colonies in the study area have been captured routinely since 1993 and currently, most birds are marked with numbered metal rings and unique colour combinations for visual assessment, and the annual breeding of all experimental colonies is monitored in detail (Fortuna et al. 2021). Birds are captured yearly and morphological measurements and a blood sample for sexing and genotyping are taken (D’Amelio et al. 2024).

###  Experiment

#### Experimental subjects and colonies

The experiment was run during Oct-Feb 2017/2018 and Oct-Jan 2019/2020. Experimental pairs were chosen with the aim of maximizing the number of groups that had helpers, as these are the focus of the current experiment. For this reason, ahead of the experiment, we analysed videos taken during rearing and selected groups with more than two birds attending the offspring. We tested 123 breeding groups, 74.8% (92) of which had helpers; the mean group size was 3.62 ± 1.42 (*N* = 123, mean ± SD). There was a total of 201 helpers of which 55 were females and 142 males (4 unsexed individuals were excluded from the analysis). The age distribution of these helpers is shown in suppl. Fig. 1. To reduce variability between nests, we aimed to target groups where the oldest chick was 8 or 9 days old on day 1 of the experiment. However, all the nests with chicks of suitable age (over 4 days old, see later) present at the colony at the moment of the experiment were also included, including nests without helpers. This resulted in an average chick age of 10.3 ± 2.5 (*N* = 123, mean ± SD) days at the beginning of the experiment, distribution shown in suppl. Fig. 2.

#### Playbacks

We aimed to mimic the presence of novel individuals in the colony to measure whether their presence would affect the feeding behaviour of individuals within the groups cooperating to raise offspring. To this end, we simulated the presence of novel individuals by playing back vocalisations of birds not living in the target colony: (a) an unknown female, (b) an unknown male and (c) an individual from a different species (our control sound; ring-necked dove *Streptopelia capicola*, a common species in the area). Our playback included contact and arrival calls which are commonly produced by birds flying to the colony and within the colony tree (Collias and Collias 2004). Individual and sex recognition through vocalisations, even short ones, is common in many species including most birds studied so far (Volodin et al. 2015; D’Amelio et al. 2017; Elie and Theunissen 2018), to the point that vocal distinctiveness appears to be a trait shared by all vocalizing species (Terry et al. 2005). Moreover, in some species, vocalisations can even signal kin relationships (Sharp et al. 2005; McDonald and Wright 2011). The commonality of sex recognition in vocalizations, combined with the monomorphic nature of sociable weavers led us to rely on the assumption that sociable weavers are able to recognize the broadcasted individuals as unknown individuals, and possibly identify their sex.

Each playback consisted of the two types of vocalisations, arrival and contact calls combined (suppl. Fig. 3). These vocalizations are sexually distinct particularly in their duration and frequency parameters (FR et al., unpublished data, examples provided as supplemetary material). The vocalisations used during the experiment were recorded at five colonies during the breeding seasons of 2014, 2015 and 2016 using a Marantz PMD 661 numeric recorder (sampling rate: 44.1 kHz) connected to a Sennheiser MKH70 directional microphone (frequency response: 50 Hz to 20 kHz ± 1dB) equipped with a foam windscreen. Birds were recorded opportunistically when flying back to the colony, on the tree or the colony structure by an experimenter hidden in a tent with an open net on each side allowing vision at 360° around the tent. The tent was settled 5–10 m from the colony and installed at least one day before the recording session to avoid disturbance. The recorded bird was identified visually through its colour rings, either directly using binoculars, or a posteriori by inspecting video recordings. All sound files were .wav files with a bit depth of 16 and a sampling rate of 44.1 kHz. The amplitude was normalized. In season 2019/2020 one extra male was recorded before the beginning of the experiment because in 2017/2018 one recorded (out of three) male was mislabelled and was in fact a female. As a consequence, 20 breeding groups in 2017/2018 were tested with two females, the control and no male playback. In total, we used recordings from 9 sociable weavers (6 females, 3 males) and 4 doves, which respectively simulated the presence of an unknown female, an unknown male and the control sound. All colonies are built in acacia trees surrounded by herbaceous vegetation, so we do not expect sound transmission to be different between the different colonies either for playback or recording of sample calls.

#### Experimental design

Breeding groups were tested for three consecutive days (schematic drawing in suppl. Fig. 4), using one stimulus each day (i.e., unknown male, unknown female or control), unless weather conditions, such as rain or strong wind, prevented it (in which case the trial would be postponed to the following day). In addition, tests were interrupted in the presence of gabar goshawks (*Micronisus gabar*), which are sociable weavers’ main adult predators, as their presence causes sociable weavers to flee inside the colony chambers, disrupting feeding behaviour (PBDA, pers. obs.).

The observation tent and the speaker box, an open container where the speaker was positioned just prior to the trials, were placed at the colony at least one day before the playback started, for habituation. The speaker box was placed on the trunk of the colony tree at a height of 2–3 m and between 2 and 3 m from the colony. For each trial, we arrived at the colony 15–30 min after sunrise and set up the speaker and cameras under the nest(s) of each focal breeding group to record the number of feeding visits to the nest by each individual. During this operation, all birds were flushed from the colony and playbacks were started by the experimenter when at least 5 individuals returned to the colony.

The order of the stimuli (i.e., unknown male, unknown female and control) was pseudorandomized for each colony and trial, and the playback stimuli were randomly assigned to each colony but ensuring that no playbacks of birds from the focal colony were played there (i.e., all playbacks were of unknown individuals). The playback consisted of an arrival call followed by four contact calls, spaced by 30 s of silence and repeated twice: this full pattern was then repeated after 30 s. Then, every four minutes, we played back the arrival call followed by the contact calls. After 10 times, therefore around 50 min of playback, we added 10 min of silence and concluded scoring the feeding visits (the total duration of each trial was 1 h; playback design scheme, suppl. Fig. 3 C). All the calls used for one stimulus belonged to the same individual.

In 2017/2018, vocalisations were played back from a CONTRACT series CT15 120-watt wall mounted speaker with a usable frequency range of 85–21,000 Hz and an impedance of 8 Ω. We played back the .wav sound files from either an iPhone 6 or a modified Western Rivers Nite Stalker. Playback peak amplitude for all calls was 53 dB at 3 m, a natural volume for all playback calls. In 2019/2020 calls were played back with an Anker loudspeaker (Soundcore Motion+), with a peak amplitude of 80 dB at 1 m.

### Feeding rate quantification and helpers-breeders assignment

Feeding rate was quantified by scoring the number of nest entrances from the video recordings by observers blind to the treatment. Each bird visiting a nest at least once was taken into account. The identity of the individuals was determined by the colour combinations of their leg rings. Birds’ entrances where scorers determined food was not carried were not recorded (e.g., when bringing nest material for nest building). When a bird was not detected feeding during the trial but was present during the experiment (e.g., seen carrying nesting material, seen during a different trial, etc.), we scored the feeding rate as ‘NA’ instead of 0. We made this decision because (i) some individuals might be away from the nest for more than one hour and (ii) helpers in this species can join the group at different stages of the breeding period (unpublished data) and therefore missing a playback day might be unrelated to the playback type.

Determining whether a bird was a breeder vs. helper was done by a combination of video and genetic analyses. Briefly, within the breeding group, we determined the ‘breeder’ or ‘helper’ status by integrating genetic parentage analysis with information about age, breeding history and the genetic relationships within the breeding group (for a detailed explanation see D’Amelio et al. 2024). Although extra-pair paternity is rare in this species (an estimated 6.4% of all clutches; D’Amelio et al. 2024), if a male breeder (i.e. social father - determined using the criteria described in D’Amelio et al. 2024) was not the biological father of all the offspring (~ 6.5% of the clutches in this experiment) he was still considered the male breeder in our analysis. The behaviour combined with historic breeding data and genetics allowed us to determine with high certainty which male and female were the breeders and which were the helpers. Only one helper might have sired one chick of the brood it was helping (uncertain genetic results), removing the helper from the analysis did not alter the results (data not shown).

### Statistical analysis

All analyses were run with R 4.1.2 (R Core Team 2024). The main aim of the analyses was to determine whether the playbacks influenced the helpers’ feeding rates. The model was designed a priori following the experimental design and including all the factors that might influence provisioning rate differently between the different experimental days and no model reduction was conducted. To study variation of feeding rate, we used a generalized linear mixed model with a Poisson link function (package lme4, Bates et al. 2015). Before interpreting the results, we checked model fitting graphically by plotting the residuals’ distribution and residuals vs. predicted values. We did not have any cases of meaningful deviation from model assumptions. As explanatory variables, we included three categorical variables: the type of playback (3 levels: male, female and control), the class of individual feeding the nestlings (2 levels: parent and helper), sex (2 levels: male and female), and the three way interaction between these variables. In addition, we added factors that are known to influence feeding rates and could differ between experimental days: time since sunrise, number of chicks in the nest (this was verified at the end of each trial), age of the chicks and temperature and wind during the trials. All continuous variables were scaled and centred to allow comparing between them (Schielzeth 2010). Furthermore, we added as factors the day of the trials (3 levels: Day 1–3) and the playback order (6 levels, corresponding to all the possible combinations of the treatments’ order). To take into account any variation due to playback exemplar we added the playback file ID (*N* = 13) as a random effect. To take into account pseudoreplication, we included bird ID for each breeding attempt (*N* = 441) as a random factor. Because birds can have different classes during different breeding attempts, we did not want to bias the estimate of one class or the other by excluding individuals who we had already sampled in different attempts as we have not studied the repeatability of provisioning behaviour. Therefore, for birds present in the study in different breeding attempts/seasons (*N* = 49), we considered two IDs. The results of this approach are qualitatively identical to one where original bird ID is considered, or replicates removed (not shown). Bird ID was nested within nest ID (*N* = 121), nested within colony ID (*N* = 25), and nested within season (*N* = 2).

We ran a follow up analysis for male helpers only, where we split them into two groups: ones too young to breed (< 237 days, the age of the youngest male breeder in our dataset) and the older ones. This resulted in a dataset of 142 birds, of which 29 were younger than our youngest male breeder and 113 were older. The model had the exact same structure but without sex and breeder classes and adding sexual maturity (2 levels) in the interaction with playback type. For both models we ran post-hoc tests using the package emmeans (Lenth 2024) to contrast all the possible combinations of playback type class of individual feeding the nestlings and their sex.

## Results

There were no significant differences between days of the experiments and between the different playback order combinations (Supp. Table 1), meaning that we did not find a general habituation to the playback and there were no order effects of the playback, hence the rest of the results did not suffer from these methodological biases.

**Fig. 1:**
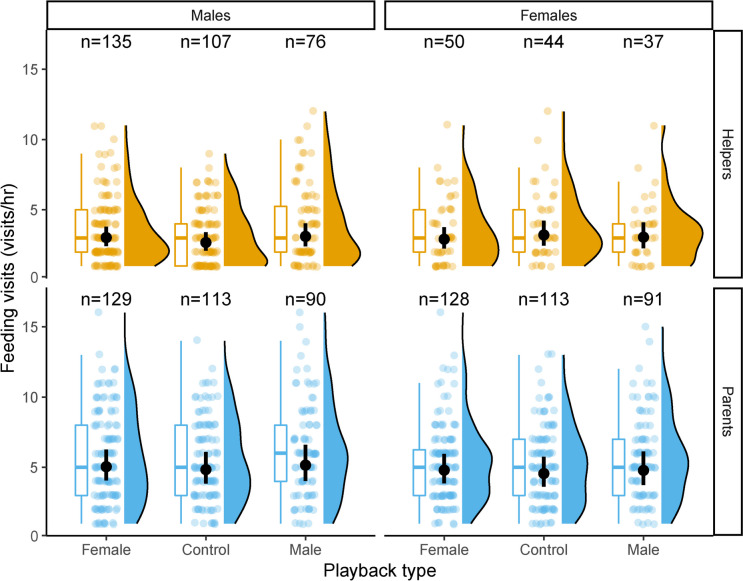
Total number of feeding visits for each of the three experimental treatments in females (right) and males (left) for helpers (top) and parents (bottom). For each facet, the results of three experimental treatments are reported (i.e., female, control, and male playbacks). For each treatment, we report the model estimate and 95% confidence interval (the black dots and bars, respectively) and the raw data, including their distribution on the right and the boxplot on the left (boxes indicate the inter quartile range (IQR), with the central line depicting the median and the whiskers extending to 1.5*IQR). The sample sizes of the birds analysed of each class, sex and treatment are reported at the top of each group

The feeding rate of the tested birds was not significantly influenced by the playback treatments in any combination of parents/helpers and sex (Fig. 1, model results in Supp. Table S1, post-hoc tests between groups, all *p* > 0.75, Supp. Table S2).

As expected, the feeding rate was positively correlated with brood size (estimate ± SE, 0.19 ± 0.02, z = 7.837, *p* < 0.001) and age of the chicks (0.07 ± 0.03, z = 2.404, *p* = 0.016). We also found that parents fed significantly more than helpers (0.35 ± 0.10, z = 3.483, *p* < 0.001).

**Fig. 2:**
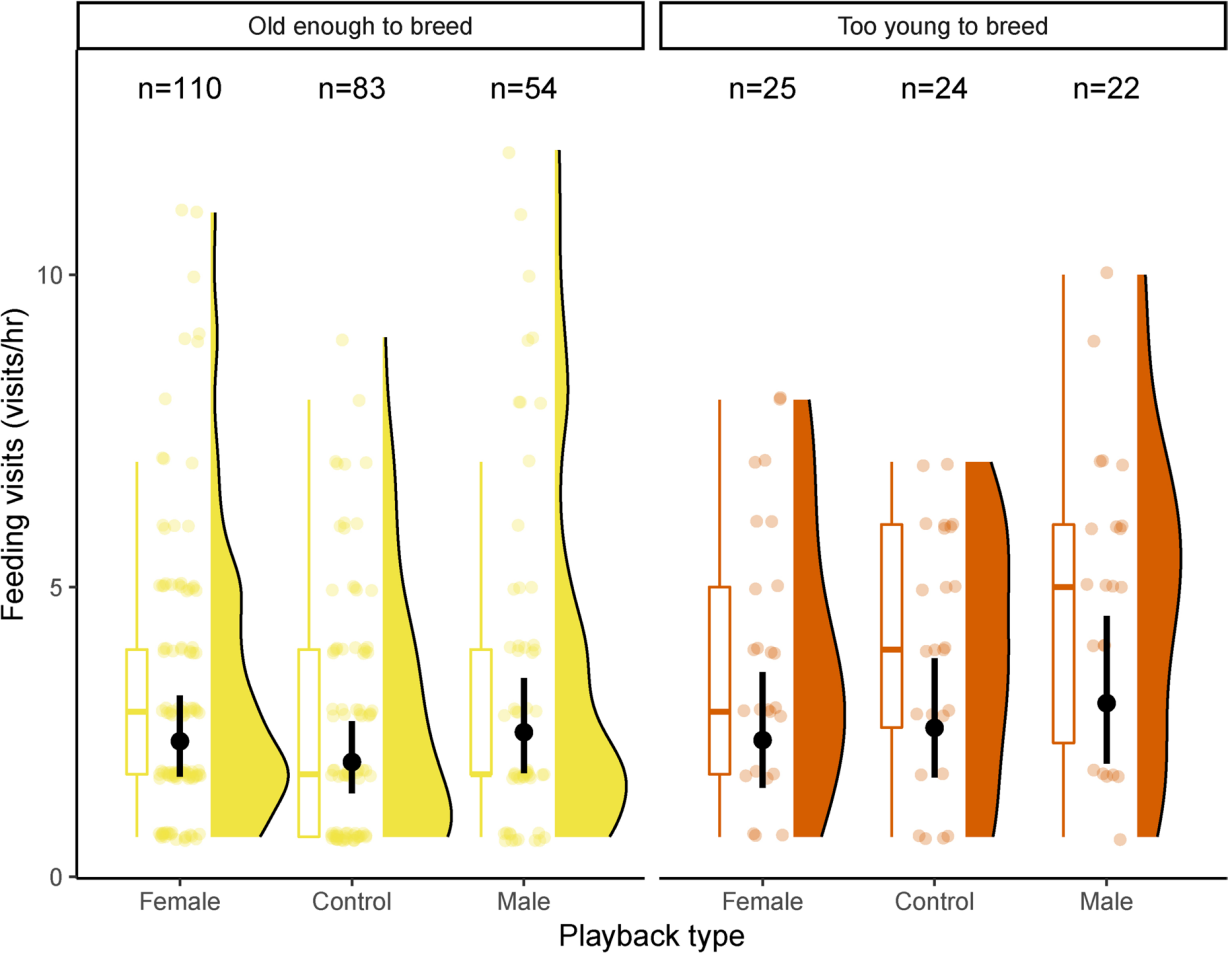
Total number of feeding visit for each of the three experimental treatments in male helpers old enough to breed (coloured in yellow, age over 237 days) vs. too young to breed (coloured in red, age under 237 days). For each facet, the three experimental treatments are reported (i.e., control, female and male playbacks). For each treatment, we report the model estimate and 95% confidence interval (black dots and segments) and the raw data, including their distribution on the right and the boxplot on the left (boxes indicate the inter quartile range (IQR), with the central line depicting the median and the whiskers extending to 1.5*IQR). The sample sizes of the birds analysed of each age group, sex and for treatment are reported at the top of each group

In the second analysis, we found that among male helpers, age class did not significantly influence feeding rate: there were no significant differences between the playback treatments in either class of male helpers (i.e., too young to breed and old enough to breed, Fig. 2, model results in Supp. Table S3, all post-hoc tests between groups *p* > 0.16, Supp. Table S4).

## Discussion

We tested whether the provisioning rate of sociable weavers changed when we simulated the arrival and presence of unknown individuals at the colony. Male helpers were expected to feed chicks more in the simulated presence of an unknown female if provisioning rate was a signal intended for potential breeding partners. Contrary to our expectation, nestling provisioning rate was not associated with the type of playback used for either sex or class (i.e., breeders and helpers). In particular, we found no increase in male helpers’ provisioning rates when we simulated the presence of an unknown female at the colony. Moreover, both young and sexually mature male helpers had very similar provisioning rates, and these were not associated with any playback type. These results suggest that the number of nestling provisioning visits is not a signal used in the context of mate acquisition/attraction of a potential sexual partner.

Our results are in agreement with experimental studies in other cooperative bird species that investigated whether chick provisioning had signalling characteristics (McDonald et al. 2008b; Nomano et al. 2013; Koenig and Walters 2016), keeping open the question of whether benefits other than the indirect benefit associated with kin selection are present in this bird species (Wright and McDonald 2016). Humans, thus, remain the only animal where it has been shown experimentally that males increase cooperation in the presence of potential sexual partners (i.e. sexual benefits, see introduction). Nevertheless, the presence of audience effects during cooperative behaviours supporting the existence of a reputation-based system where good cooperators increase the probability of receiving social benefits has been found in cichlid fish (Kingma et al. 2014), cleaner fish (Pinto et al. 2011) and humans (Van Vugt and Iredale 2013; Raihani and Smith 2015). However, in other species, cooperative acts that possibly result in social benefits were not found to have signalling characteristics. For example, in Norway rat, *Rattus norvegicus*, the presence of an audience did not influence their propensity to pull food towards a social partner (Schweinfurth and Taborsky 2016). In Common marmosets *Callithrix jacchus*, the presence of an audience suppressed helping behaviour such as food sharing with immatures (Brügger et al. 2018). This raises the question: what are the common features of systems where cooperative behaviours act, or do not act, as explicit signals? For example, mate choice benefits associated with cooperation may be primarily driven by male-male competition rather than female choice (Nomano et al. 2013), making a signal intended for a female irrelevant.

To better predict, in sociable weavers and other species, whether and how cooperative feeding could be used as a signal in a breeding/mate attraction context, future studies should aim to better understand pair formation and the transition from helping to breeding. Focusing on mate choice (who helpers will pair with) and timing (when helpers pair up) is essential to conclusively identify the target of any mating-related signals. In addition, to determine if provisioning rate can be a signal it should be determined if it conveys reliable information. This requires quantifying what factors influence provisioning rate (e.g., individual condition), how variable this behaviour is (e.g., within and across breeding season and attempts), and how it predicts aspects of breeding success (e.g., investment in future breeding attempts) (MacColl and Hatchwell 2003; Bergmüller et al. 2010; English et al. 2010).

It is important to also point out that simulating the presence of a prospecting individual is not always straightforward. In our experiment, as our playback occurred at fixed times and not in response to a specific individual, we cannot completely rule out that our playback did not effectively simulate the presence of a potential mate to all of the individuals observed. In the future, using emerging technological techniques, experiments could conduct more targeted playbacks. For example, by using automated RFID to trigger playbacks (Lendvai et al. 2015), researchers could target specific individuals to ensure the playback is received by them. In addition, hidden or more realistic sound sources, such as stuffed animals or 3D printed models might help to limit possible habituation, and increase realism. Moreover another factor that may have influenced the response to the playback in our experiment is the relatedness of the helpers to the offspring, as unrelated helpers which do not receive indirect benefits, may use provisioning as a signal more than related ones (Wright 2007; Doutrelant et al. 2011; Zöttl et al. 2013). Experiments using a targeted approach could focus on unrelated helpers only (McDonald et al. 2009), further refining the experimental questions.

Similarly, to ensure that the intended receiver actually receives a playback, producing naturally sounding calls can be difficult to achieve in an experimental situation, especially for calls that have only had cursory study. For example, while the general use of arrival and contact calls has been established (Collias and Collias 2004), and sex and identity are often found to be encoded in bird calls (Terry et al. 2005; Volodin et al. 2015) whether individual and sexual characteristics of the sound are perceived or attended to by receivers in sociable weavers, remains unknown. Additionally, more context specific calls or production may be used as well. For example, it is possible that prospecting immigrant females use specific vocalisations or vocalisations produced at a specific rate to advertise their presence or receptivity to a mate, and that the arrival and contact calls we used (calls of individuals returning to their home colony) are used only by individuals established in the colony. In summary, to ensure that not only the correct call production is used in future experiments, but also that the prospecting behaviour itself is well reproduced, future studies should first establish how the process of female dispersal and integration in the colony occur. Specifically, whether a series of behavioural stages is needed before the newcomers are integrated into the colony group (Jungwirth et al. 2015) and can be perceived by the colony members as potential partners.

Finally, while provisioning rate is a useful choice as a potential signal to investigate, other aspects of cooperative feeding that are less often investigated might play a potential role in signalling, such as its advertisement through vocalisations and/or prey size and display. For instance a previous study suggested sociable weavers’ decisions regarding the size of the prey they bring to provision young and the manner in which they display their helping behaviour varied with both the presence of an audience and environmental conditions (Doutrelant and Covas 2007), however provisioning rate was not tested. In addition, nestling provisioning rate in itself may not be used by the potential receivers as a signal because the number of feeding visits may be difficult to quantify for the observers and simpler signals might be preferred (Roberts 1998; Clutton-Brock et al. 2005).

From our results, we concluded that our playback experiment did not affect the provisioning rate of the helpers and that it is unlikely that, in sociable weavers, the variation in the feeding visits is influenced by sexual selection. However, the display of helping behaviour shown by sociable weavers (Doutrelant and Covas 2007) remains a compelling behaviour to suggest that cooperative behaviour may have evolved a secondary signalling function in a mate choice context. By continuing to explore displays of helping behaviour through more targeted experiments, more naturalistic playbacks, a better understanding of the many variables that could impact specific cooperative behaviours, and a complete understanding of the behaviour and process of mate choice in cooperative species, future experiments can elucidate the role that cooperation may have on intraspecific communication and mate choice.

## Supplementary information

Below is the link to the supplementary material.

ESM 1 (DOCX 1.07 MB)

ESM 2 (WAV 227 KB)

ESM 3 (WAV 150 KB)

ESM 4 (WAV 264 KB)

ESM 5 (WAV 199 KB)

ESM 6 (WAV 19.7 KB)

ESM 7 (WAV 20.8 KB)

ESM 8 (WAV 14.4 KB)

ESM 9 (WAV 18.7 KB)

## Data Availability

Data and scripts are publicly available at: 10.6084/m9.figshare.27934380.
