# Supplementary material for: Is provisioning rate of parents and helpers influenced by the simulated presence of novel individuals?
Source: Behav Ecol Sociobiol. 2025 Jan 15;79(1):14. doi: 10.1007/s00265-024-03548-2 (PMC11735550; doi:10.1007/s00265-024-03548-2)
Supplement: Supplementary file 1 — (DOCX 1.07 MB) [file 265_2024_3548_MOESM1_ESM.docx]

**Appendix for:**

**Is provisioning rate of parents and helpers influenced by the simulated presence of novel individuals?**

**Published in: Behavioral ecology and sociobiology**

By:

D’Amelio B. Pietro^1,2,3,4^, Carlson V. Nora^3,5^, Tognetti Arnaud^6^, Sentís Marina^7^, Silva R. Liliana^8,9^, Rybak Fanny^3^, Covas Rita^2,8,9^, Doutrelant Claire^1,2^

^1^ Centre d’Ecologie Fonctionnelle et Evolutive, CNRS, Univ Montpellier, EPHE, IRD, Montpellier, France

^2^ FitzPatrick Institute of African Ornithology, DSI-NRF Centre of Excellence, University of Cape Town, Rondebosch 7701, South Africa

^3^ Université Paris-Saclay, CNRS, Institut des Neurosciences Paris-Saclay, 91400, Saclay, France

^4^Consiglio Nazionale delle Ricerche—Istituto di Ricerca sulle Acque (CNR-IRSA), Brugherio, Italy

^5^ Juanes Lab, Department of Biology, University of Victoria, Victoria, BC

^6^ CEE-M, CNRS, INRAE, Institut Agro, University of Montpellier, Montpellier, France

^7^ Terrestrial Ecology Unit, Department of Biology, Ghent University, Ghent, Belgium

^8^ CIBIO-InBio, Centro de Investigação em Biodiversidade e Recursos Genéticos, Laboratório Associado, University of Porto, Campus Agrário de Vairão, 4485-661 Vairão, Portugal

^9^ BIOPOLIS Program in Genomics, Biodiversity and Land Planning, CIBIO, Campus de Vairão, 4485-661 Vairão, Portugal

Corresponding author:

Pietro B. D'Amelio

[pie.damelio@gmail.com](mailto:pie.damelio@gmail.com)

This document contains:

4 Figures

4 Tables

The scripts for the figures and analyses can be found:

10.6084/m9.figshare.27934380


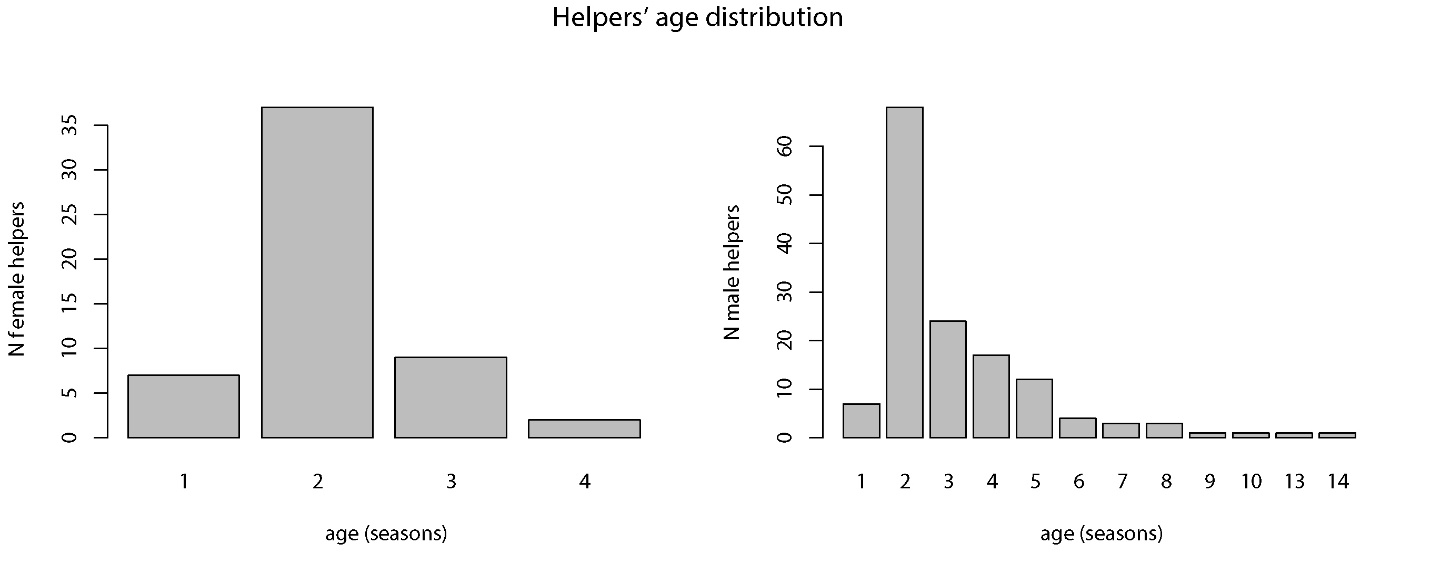


# Supplementary figure 1

Bar plot of the age distribution of helpers across seasons, females (N=55) on the left and males (N=142) on the right. Season = 1 means born in the same season that they are helping (i.e., less than 1 year old).


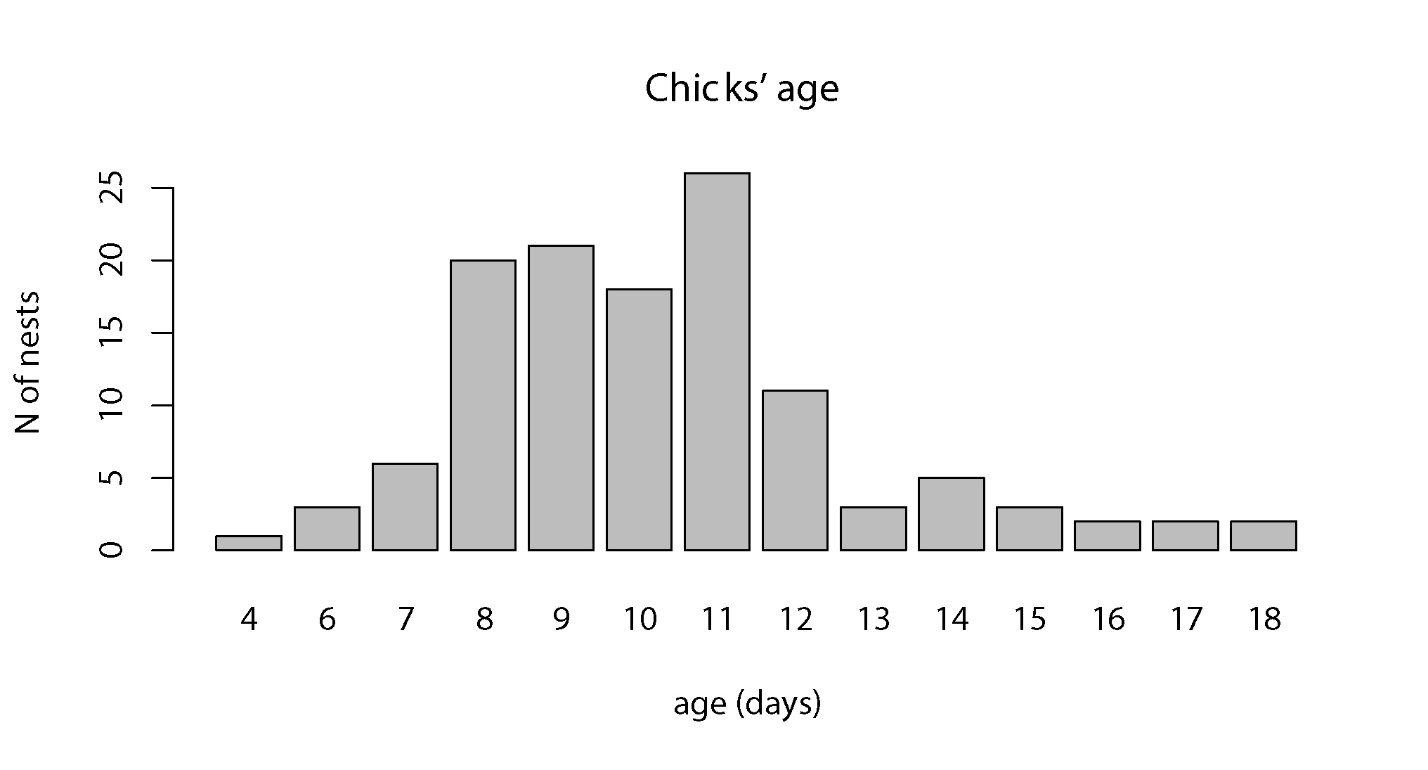


# Supplementary figure 2

Bar plot of the chicks’ age in days. One value per nest, the age of the first hatched chick, is plotted (N=123).


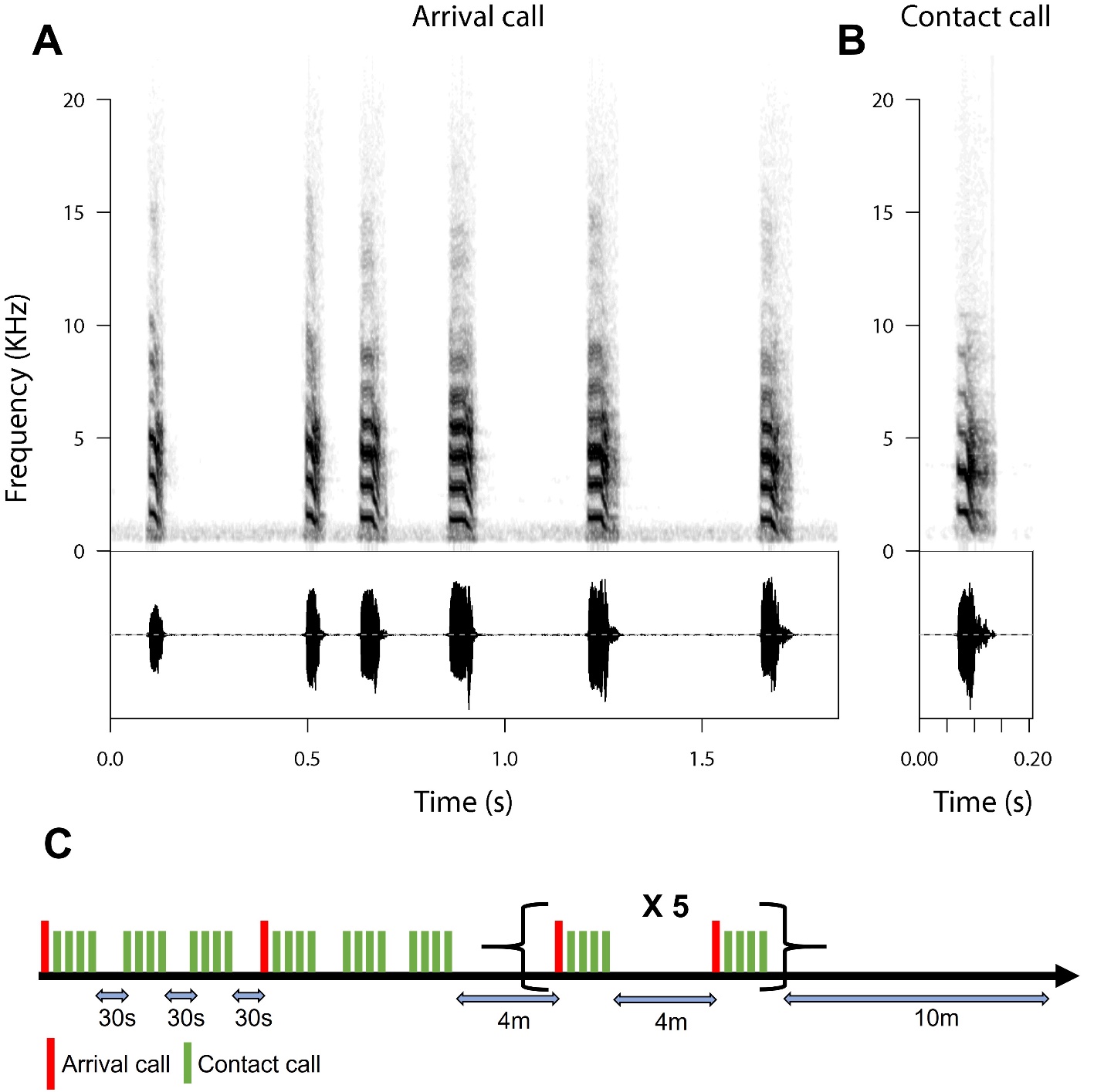


# Supplementary Figure 3

Spectrograms and oscillograms illustrating a representative example of an arrival call (A) and a contact call (B). Spectrograms were generated with the ‘soundgen’ R package (Anikin, 2019), using the original audio files with sampling rate of 44.1 kHz, window type Gaussian, length of fast Fourier transforms (FFT) windows 50 ms, and 70% overlap between successive FFT frames. Oscillograms below each spectrogram represent the non-normalized amplitude envelopes.

At the bottom (C) there is a graphical representation of the playback track design. The intervals between different sets of calls are not in scale, s= seconds, m=minutes. The portion within curly brackets is repeated 5 times.


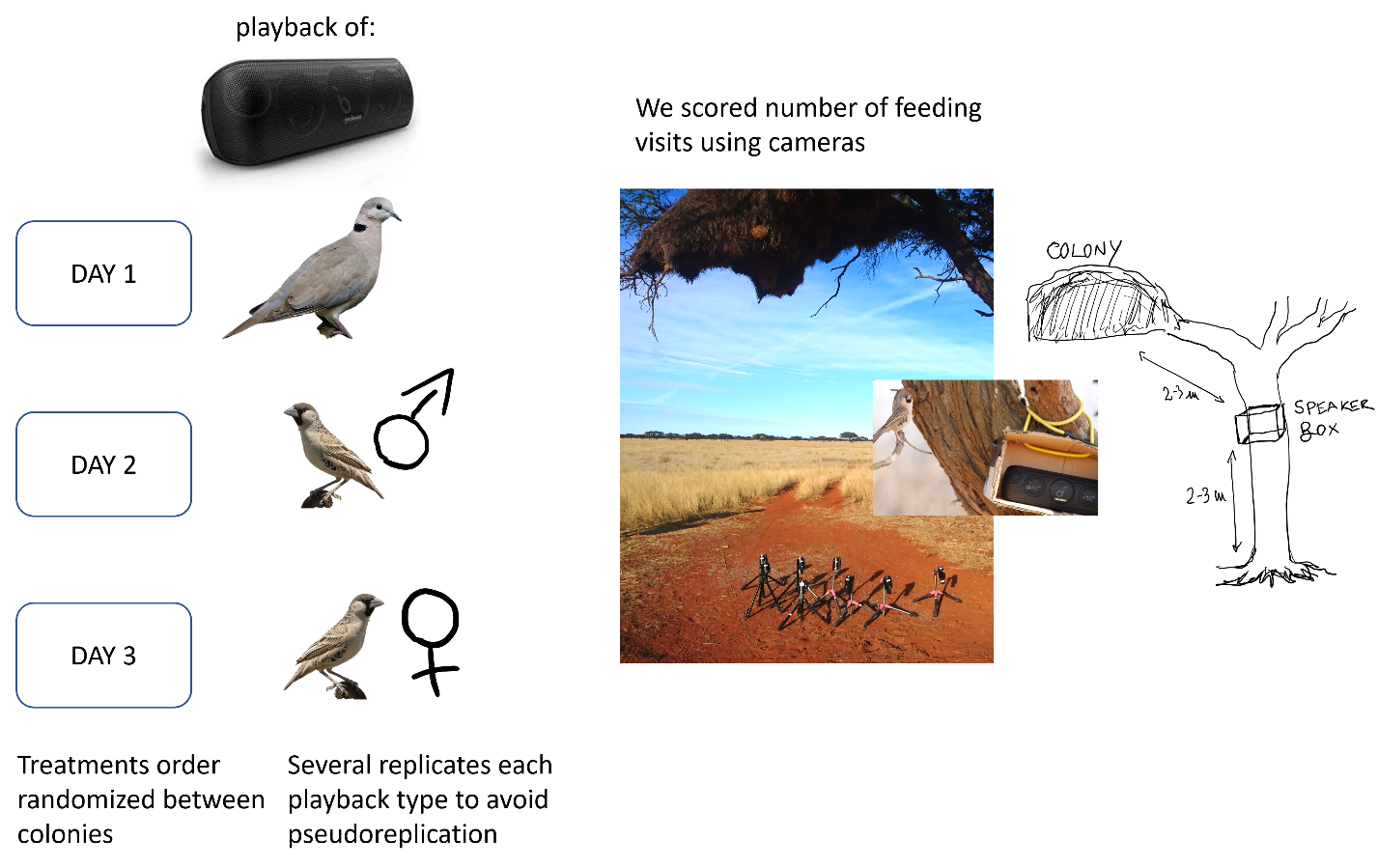


# Supplementary Figure 4

Visualization of the experiment design and set up. One playback stimulus was played each day from a loudspeaker placed on the colony tree, 2-3 meters from the colony structure and 2-3 meters high. Birds sometimes approached the loudspeaker showing interest towards the stimulus as depicted in the picture in the center.

Model structure of the main model as coded in R (full R script provided):

mod<- glmer (Feeding visits ~ Playback type * Class * Sex +

Time since sunrise + Number of chicks + Age of the chicks + Temperature + Wind + Recording day + Playback order + (1|Playback file) + (1|Season/Colony/Nest/BirdID),

data=dat, family ="poisson", glmerControl(optimizer ='optimx', optCtrl=list(method='nlminb')))

# Supplementary Table 1

Model summary of the results of the linear mixed model testing whether the playbacks influenced feeding rates. Shown are regression coefficients, their 95% confidence intervals and the correspondent P-value, for each of the fixed factors. All continuous variables were scaled (for each variable’s value the variable’s mean is subtracted, and the result is divided by the variable’s standard deviation) and therefore the estimates are directly comparable. The reference level (i.e., the one used as comparison to and therefore not present in the table) is control playback, females, helpers, recording day 1 and playback order 5 (chosen because of alphabetic order). The random effects sample sizes are also reported for each group.

For a visualization of the fitted effects, see Figure 1 in the main text.

|  | | | **Feeding visits** | |
| --- | --- | --- | --- | --- |
| *Predictors* | *Log-Mean* | *CI* | | *p* |
| (Intercept) | 1.17 | 0.89 – 1.44 | | **<0.001** |
| Playback type [F] | -0.10 | -0.37 – 0.18 | | 0.484 |
| Playback type [M] | -0.05 | -0.36 – 0.26 | | 0.751 |
| Class [parent] | 0.35 | 0.15 – 0.55 | | **<0.001** |
| Sex [M] | -0.18 | -0.39 – 0.03 | | 0.089 |
| Time since sun rise | 0.01 | -0.06 – 0.07 | | 0.833 |
| Number of chicks | 0.19 | 0.14 – 0.23 | | **<0.001** |
| Age of the chicks z | 0.07 | 0.01 – 0.12 | | **0.016** |
| Temperature | 0.05 | -0.01 – 0.10 | | 0.079 |
| Wind | -0.00 | -0.04 – 0.04 | | 0.910 |
| Recording day [2] | 0.07 | -0.01 – 0.15 | | 0.069 |
| Recording day [3] | 0.06 | -0.03 – 0.16 | | 0.202 |
| Playback order [four] | -0.04 | -0.22 – 0.15 | | 0.704 |
| Playback order [one] | -0.04 | -0.19 – 0.11 | | 0.568 |
| Playback order [six] | -0.00 | -0.20 – 0.20 | | 0.995 |
| Playback order [three] | 0.16 | -0.02 – 0.35 | | 0.084 |
| Playback order [two] | -0.05 | -0.25 – 0.14 | | 0.580 |
| Playback type [F] * Class [parent] | 0.15 | -0.10 – 0.40 | | 0.233 |
| Playback type [M] * Class [parent] | 0.10 | -0.17 – 0.36 | | 0.471 |
| Playback type [F] * Sex[M] | 0.22 | -0.04 – 0.49 | | 0.098 |
| Playback type [M] * Sex[M] | 0.20 | -0.09 – 0.49 | | 0.172 |
| Class [parent] * Sex [M] | 0.24 | -0.01 – 0.49 | | 0.056 |
| (Playback type [F] * Class [parent]) * Sex [M] | -0.23 | -0.54 – 0.07 | | 0.138 |
| (Playback type [M] * Class [parent]) * Sex [M] | -0.18 | -0.52 – 0.15 | | 0.277 |
| **Random Effects** | | | | |
| σ^2^ | 0.22 | | | |
| _BirdID: (Nest:(Colony:Season)) (N=441)_ | 0.06 | | | |
| _Nest: (Colony:Season) (N=51)_ | 0.00 | | | |
| _Colony:Season (N=14)_ | 0.01 | | | |
| _Playback_file (N=13)_ | 0.02 | | | |
| _Season (N=2)_ | 0.01 | | | |
| Observations | 1113 | | | |
| Marginal R^2^ / Conditional R^2^ | 0.316 / NA | | | |

# Supplementary table 2

Contrasts between estimated marginal means for each experimental group. The playback treatments are summarized as follows, C=control, F=female, M=male. Sex refers to the sex of the bird of which the feeding rate was measured.

We applied Bonferroni correction to account for multiple testing.

| **Contrast** | **Sex** | **Class** | **Estimate** | **SE** | **z-ratio** | **P-value** |
| --- | --- | --- | --- | --- | --- | --- |
| C - F | F | helper | 0.098671373 | 0.1409070 | 0.70025873 | 1.0000000 |
| C - M | F | helper | 0.050227200 | 0.1585243 | 0.31684220 | 1.0000000 |
| F - M | F | helper | -0.048444173 | 0.1530803 | -0.31646241 | 1.0000000 |
| C - F | M | helper | -0.123773288 | 0.1139646 | -1.08606760 | 0.8323473 |
| C - M | M | helper | -0.150711880 | 0.1310838 | -1.14973699 | 0.7507566 |
| F - M | M | helper | -0.026938592 | 0.1225602 | -0.21979888 | 1.0000000 |
| C - F | F | parent | -0.050866476 | 0.1046255 | -0.48617679 | 1.0000000 |
| C - M | F | parent | -0.047730421 | 0.1195945 | -0.39910220 | 1.0000000 |
| F - M | F | parent | 0.003136055 | 0.1122967 | 0.02792651 | 1.0000000 |
| C - F | M | parent | -0.042018909 | 0.1038295 | -0.40469130 | 1.0000000 |
| C - M | M | parent | -0.063944286 | 0.1186652 | -0.53886288 | 1.0000000 |
| F - M | M | parent | -0.021925377 | 0.1113511 | -0.19690305 | 1.0000000 |

# Supplementary table 3

Model summary of the results of the linear mixed model testing whether the playbacks influenced feeding rates of male helpers. Shown are regression coefficients, their 95% confidence intervals and the correspondent P-value. For each of the fixed factors, all continuous variables were scaled (for each variable’s value the variable’s mean is subtracted, and the result is divided by the variable’s standard deviation) and therefore the estimates are directly comparable. For the random effects we also reported the sample sizes of each group.

For a visualization of the fitted effects, see Figure 2 in the main text.

|  | **Feeding visits** | | |
| --- | --- | --- | --- |
| *Predictors* | *Log-Mean* | *CI* | *p* |
| (Intercept) | 0.79 | 0.53 – 1.05 | **<0.001** |
| Playback type [F] | 0.14 | -0.03 – 0.31 | 0.111 |
| Playback type [M] | 0.20 | -0.00 – 0.40 | 0.056 |
| Age [too young] | 0.22 | -0.09 – 0.53 | 0.163 |
| Time since sunrise | 0.02 | -0.07 – 0.11 | 0.704 |
| Number of chicks | 0.12 | 0.01 – 0.22 | **0.025** |
| Age of the chicks | -0.01 | -0.12 – 0.10 | 0.865 |
| Temperature | 0.05 | -0.05 – 0.14 | 0.323 |
| Wind | 0.02 | -0.06 – 0.10 | 0.572 |
| Recording day [2] | 0.06 | -0.11 – 0.23 | 0.476 |
| Recording day [3] | 0.14 | -0.04 – 0.32 | 0.138 |
| Playback type [four] | -0.08 | -0.45 – 0.29 | 0.674 |
| Playback type [one] | 0.20 | -0.07 – 0.47 | 0.141 |
| Playback type [six] | 0.28 | -0.04 – 0.60 | 0.084 |
| Playback type [three] | 0.20 | -0.15 – 0.55 | 0.258 |
| Playback type [two] | 0.08 | -0.23 – 0.39 | 0.625 |
| Playback type [F] * Age [too young] | -0.22 | -0.55 – 0.12 | 0.207 |
| Playback type [M] * Age [too young] | -0.06 | -0.41 – 0.29 | 0.728 |
| **Random Effects** | | | |
| σ^2^ | 0.28 | | |
| _BirdID: (Nest:(Colony:Season)) (N=142)_ | 0.11 | | |
| _Nest:(Colony:Season) (N=39)_ | 0.01 | | |
| _Colony:Season (N=14)_ | 0.00 | | |
| _Playback_file (N=13)_ | 0.00 | | |
| _Season (N=2)_ | 0.00 | | |
| Observations | 318 | | |
| Marginal R^2^ / Conditional R^2^ | 0.174 / NA | | |

# Supplementary table 4

Post hoc tests for the male helpers only model. Contrasts between estimated marginal means for each experimental group. The playback treatments are summarized as follows, C=control, F=female, M=male. “could_breed” refers to male helpers older than 257 days (the youngest breeder in our population), “too_young” refers to the younger male helpers. We applied a Bonferroni correction to account for multiple testing

| **Contrast** | **Age - categorical** | **Estimate** | **SE** | **z-ratio** | **P-value** |
| --- | --- | --- | --- | --- | --- |
| C - F | could_breed | -0.14107670 | 0.08852407 | -1.5936535 | 0.3330410 |
| C - M | could_breed | -0.19643017 | 0.10263255 | -1.9139168 | 0.1668924 |
| F - M | could_breed | -0.05535347 | 0.09639531 | -0.5742341 | 1.0000000 |
| C - F | too_young | 0.07405750 | 0.14969938 | 0.4947082 | 1.0000000 |
| C - M | too_young | -0.13491281 | 0.14732827 | -0.9157292 | 1.0000000 |
| F - M | too_young | -0.20897031 | 0.14547392 | -1.4364795 | 0.452597 |

# Model reduction approach:

We demonstrate below that the conclusion reached would be exactly the same by performing stepwise model selection. Thus, we are confident that our results are solid and do not depend on the statistical method used. We prefer to keep the current statistical method as it is the one recommended for reproducible research and generally currently advised by the great majority of the statistical community. These analysis are in the script to be published alongside the paper.

**Likelihood ratio test**

| **Predictor removed** | **Mod Name** | **DF** | **LogLik** | **Chisq** | **Pr(>Chisq)** |
| --- | --- | --- | --- | --- | --- |
| FULL model | 1 | 29 | -2430 |  |  |
| Class (helper/breeder) | 2 | 23 | -2496 | 131 | < 2e-16 |
| Sex | 3 | 23 | -2433 | 126 | < 2e-16 |
| Playback type (male/female/control) | 4 | 21 | -2432 | 1.3 | 0.50339 |
| Time since sunrise | 5 | 28 | -2430 | 3.7 | 0.81250 |
| Number of chicks | 6 | 28 | -2458 | 56 | < 2e-16 |
| Day of the chicks | 7 | 28 | -2433 | 50 | < 2e-16 |
| Temperature | 8 | 28 | -2432 | 2.4 | < 2e-16 |
| Wind | 9 | 28 | -2430 | 3 | < 2e-16 |
| Recording day | 10 | 27 | -2432 | 3.3 | 0.06933 |
| Playback order | 11 | 24 | -2433 | 3.2 | 0.35866 |

The stepwise model selection shows that, as in the model included in the manuscript, playback, time since sunrise, recording day, and playback order did not have a significant effect. When we ran the reduced model (excluding the non-significant factors except playback) our results agree with the model included in the manuscript (qualitatively they are the same). See below for the post-hoc estimates and P-Values for the comparisons between the different playbacks in each class of birds of the reduced model (for the package used see main text)

sex = **F**, class = **helper**:

| contrast estimate | SE | df | z.ratio | p.value |
| --- | --- | --- | --- | --- |
| C – F | 0.0983 - 0.139 | Inf | 0.707 | 0.7594 |
| C – M | 0.0495 - 0.156 | Inf | 0.316 | 0.9463 |
| F – M | -0.0488 - 0.151 | Inf | -0.323 | 0.9442 |

(C=control playback, F=female playback, M=male playback)

sex = **M**, class = **helper**:

| contrast estimate | SE | df | z.ratio | p.value |
| --- | --- | --- | --- | --- |
| C – F | -0.1387 - 0.112 | Inf | -1.242 | 0.4287 |
| C – M | -0.1597 - 0.129 | Inf | -1.243 | 0.4280 |
| F – M | -0.0211 - 0.120 | Inf | -0.175 | 0.9832 |

(C=control playback, F=female playback, M=male playback)

sex = **F**, class = **breeder:**

| contrast estimate | SE | df | z.ratio | p.value |
| --- | --- | --- | --- | --- |
| C – F | -0.0598 - 0.102 | Inf | -0.587 | 0.8271 |
| C – M | -0.0551 - 0.117 | Inf | -0.472 | 0.8843 |
| F – M | 0.0047 - 0.110 | Inf | 0.043 | 0.9990 |

(C=control playback, F=female playback, M=male playback)

sex = **M**, class =**breeder:**

| contrast estimate | SE | df | z.ratio | p.value |
| --- | --- | --- | --- | --- |
| C – F | -0.0504 0.101 | Inf | -0.499 | 0.8718 |
| C – M | -0.0719 0.116 | Inf | -0.622 | 0.8080 |
| F – M | -0.0215 0.109 | Inf | -0.197 | 0.9788 |

(C=control playback, F=female playback, M=male playback)

# References

Anikin, A. (2019). Soundgen: An open-source tool for synthesizing nonverbal vocalizations. *Behavior Research Methods*, *51*(2), 778–792. https://doi.org/10.3758/S13428-018-1095-7/FIGURES/4
